# Accelerometer-Measured Patterns of Sedentary Behavior in Older Women: The OPACH Study

**DOI:** 10.1093/geroni/igab046.1312

**Published:** 2021-12-17

**Authors:** Benjamin Schumacher, Eric Hyde, John Bellettiere, Blake Anuskiewicz, Andrea LaCroix

**Affiliations:** 1 University of California, San Diego, San Diego, California, United States; 2 UC San Diego, San Diego, California, United States; 3 University of California, San Diego Herbert Wertheim School of Public Health and Human Longevity Science, La Jolla, California, United States; 4 San Diego State University, San Diego, California, United States

## Abstract

Excessive sedentary behavior (SB) is related to deleterious health outcomes. Understanding the patterns and contexts in which SB accumulates can promote healthy aging. Daily sitting time and mean sitting bout duration (MBD) were measured by triaxial accelerometers. Participants self-reported how much time they spent sitting while: watching TV, reading, using the computer, driving, working, or taking phone calls. Data were compared across aging-related characteristics. Age-adjusted sitting time (minutes/day) for 5,838 diverse (33.2% Black, 16.9% Hispanic), older women (mean age 78.7±6.7) were 577.2 for Hispanic women, 630.3 for Black women, and 632.0 for White women. Those in the lowest vs. highest physical function category had the longest MBD (16.1 vs. 11.7 minutes/bout). Watching television was the most common self-reported sedentary activity. The highest vs. lowest quartile of MBD spent, on average, 30.6 and 22.3 minutes/day watching television, respectively. This presentation will illuminate critical factors associated with sitting patterns in older adults.

